# Accelerated Evolution of Mitochondrial but Not Nuclear Genomes of Hymenoptera: New Evidence from Crabronid Wasps

**DOI:** 10.1371/journal.pone.0032826

**Published:** 2012-03-06

**Authors:** Martin Kaltenpoth, Patrice Showers Corneli, Diane M. Dunn, Robert B. Weiss, Erhard Strohm, Jon Seger

**Affiliations:** 1 Research Group Insect Symbiosis, Max Planck Institute for Chemical Ecology, Jena, Germany; 2 Department of Human Genetics, University of Utah, Salt Lake City, Utah, United States of America; 3 Zoological Institute, University of Regensburg, Regensburg, Germany; 4 Department of Biology, University of Utah, Salt Lake City, Utah, United States of America; Biodiversity Insitute of Ontario - University of Guelph, Canada

## Abstract

Mitochondrial genes in animals are especially useful as molecular markers for the reconstruction of phylogenies among closely related taxa, due to the generally high substitution rates. Several insect orders, notably Hymenoptera and Phthiraptera, show exceptionally high rates of mitochondrial molecular evolution, which has been attributed to the parasitic lifestyle of current or ancestral members of these taxa. Parasitism has been hypothesized to entail frequent population bottlenecks that increase rates of molecular evolution by reducing the efficiency of purifying selection. This effect should result in elevated substitution rates of both nuclear and mitochondrial genes, but to date no extensive comparative study has tested this hypothesis in insects. Here we report the mitochondrial genome of a crabronid wasp, the European beewolf (*Philanthus triangulum*, Hymenoptera, Crabronidae), and we use it to compare evolutionary rates among the four largest holometabolous insect orders (Coleoptera, Diptera, Hymenoptera, Lepidoptera) based on phylogenies reconstructed with whole mitochondrial genomes as well as four single-copy nuclear genes (18S rRNA, arginine kinase, wingless, phosphoenolpyruvate carboxykinase). The mt-genome of *P. triangulum* is 16,029 bp in size with a mean A+T content of 83.6%, and it encodes the 37 genes typically found in arthropod mt genomes (13 protein-coding, 22 tRNA, and two rRNA genes). Five translocations of tRNA genes were discovered relative to the putative ancestral genome arrangement in insects, and the unusual start codon TTG was predicted for cox2. Phylogenetic analyses revealed significantly longer branches leading to the apocritan Hymenoptera as well as the Orussoidea, to a lesser extent the Cephoidea, and, possibly, the Tenthredinoidea than any of the other holometabolous insect orders for all mitochondrial but none of the four nuclear genes tested. Thus, our results suggest that the ancestral parasitic lifestyle of Apocrita is unlikely to be the major cause for the elevated substitution rates observed in hymenopteran mitochondrial genomes.

## Introduction

Mitochondrial genes have been used extensively for phylogenetic studies in insects. Their generally high substitution rates render them especially useful to resolve the relationships among closely related taxa [1]. Deeper phylogenetic splits, however, are usually not well resolved in analyses based on mitochondrial genes, and the high heterogeneity in among-site rate variation may partly be responsible for the poor performance of mitochondrial as compared to nuclear genes [2]. An additional problem with mitochondrial sequences is that differences in mitochondrial evolutionary rates among insect lineages can cause long-branch attraction problems [3] that result in unrelated taxa with high substitution rates erroneously grouping together in phylogenetic trees [4]. A similar effect has been observed as a consequence of occasional reversals in the strand-specific compositional bias that is often pronounced in mitochondrial genomes [5]–[7].

Recently, the availability of an increasing number of complete insect mitochondrial genomes has initiated phylogenomic approaches that have greatly enhanced our understanding of the evolutionary relationships within and among extant hexapod orders [8]–[13]. Despite these efforts, the range of insect taxa for which complete mitochondrial genomes are available remains rudimentary, and many large families are not represented by a single sequence. This is also true for several families within the Hymenoptera, one of the largest insect orders on earth. Notably, no mitochondrial genome sequence is available for the about 8000 species of Crabronidae, although they constitute the sister group to the Apidae, a family of considerable interest due its ecological and economical importance and the wide range of social systems represented in this taxon [14].

Substitution rates of mitochondrial genomes have been found to vary substantially across insect taxa. Notably, Hymenoptera and Phthiraptera exhibit significantly elevated rates of nucleotide substitutions [11], [15]–[17], which has been attributed to the parasitic lifestyle of the extant or ancestral members of these orders [18], [19]. The usually short generation times and small effective population sizes due to frequent founder events typically found in parasitic lineages would be expected to result in elevated nucleotide substitution rates in both mitochondrial and nuclear genes [16], [20]. However, to date no detailed multi-gene study is available that compares evolutionary rates between mitochondrial and nuclear genes in parasitic versus non-parasitic insect taxa (but see [21]).

Here we report on the first complete mitochondrial genome sequence of a crabronid wasp, the European beewolf, *Philanthus triangulum* (Hymenoptera, Crabronidae). Due to their interesting natural history, *Philanthus* species have attracted considerable attention among behavioral ecologists, and their biology has been studied in detail [22]–[24]. Recently, *Philanthus* females have been found to engage in an unusual symbiosis with the actinobacterium ‘*Candidatus* Streptomyces philanthi’ [25], [26]. These bacteria are cultivated in unique antennal gland reservoirs of female beewolves [27] and transferred to the larval cocoon [28], where they provide protection against pathogenic microorganisms by producing a cocktail of antibiotic substances [29].

Using complete mitochondrial genome sequences as well as four different nuclear gene datasets, we reconstructed the phylogenetic relationships among the four largest holometabolous insect orders (Coleoptera, Hymenoptera, Diptera, and Lepidoptera), and we compared the substitution rates of mitochondrial and nuclear genes among the orders. Based on earlier studies [21], we hypothesized that the ancestral parasitic lifestyle of apocritan Hymenoptera resulted in elevated substitution rates in both mitochondrial and nuclear genomes.

## Materials and Methods

### Ethics statement

A male European beewolf was obtained from a laboratory culture that had earlier been established with field-collected animals from Würzburg and Erlangen, Germany. No specific permits were required for collecting, as *P. triangulum* is not an endangered or protected species, and the collecting localities constituted non-protected public areas.

### Mitochondrial genome sequencing, assembly and annotation

Whole genomic DNA was extracted from the thorax of the male beewolf by standard phenol-chloroform extraction [30] and stored at 4°C in 100 µl Low-TE (1 mM Tris, 0.1 mM EDTA, pH 8.0). For the PCRs, 2 µl of a 1∶10 dilution were used per 25 µl PCR mix. About half of the mitochondrial genome (7891 bp) could be amplified by using the primers C2LF2 and CBLR5 with the Peqlab MidRange PCR System according to the manufacturer's suggestions (Table S1 and S2). The remaining part was amplified in smaller fragments (Table S2). For fragments spanning the AT-rich control region, the extension temperature had to be reduced to 60°C for successful amplification [31], [32]. The amplicons were sequenced bidirectionally by primer walking on ABI 3700 or ABI 3730 instruments using the Big Dye terminator kit (for sequencing primers see Table S1).

Sequences were edited and assembled using the Phred/Phrap/Consed package [33]–[35]. The whole mitochondrial genome sequence was saved as a FASTA file, and tRNAscan-SE 1.21 was used for tRNA search and secondary structure prediction [36]. Lowering the Cove threshold to 1 yielded all of the expected tRNA genes except for the tRNA-Ser(AGN) gene that has proven difficult to find in earlier studies due to its unusual secondary structure [37]. This gene was detected by aligning other hymenopteran tRNA-Ser(AGN) genes with the *P. triangulum* mitochondrial genome, and the sequence was manually inspected and the annotation corrected based on the predicted secondary structure. The localization was confirmed by using MOSAS [38]. For annotation of protein-coding genes and rRNAs, the sequence file was imported into DOGMA [39]. All genes were checked and corrected manually on the basis of the predicted amino acid sequences and BLAST searches against available mitochondrial genomes. The rRNA genes were compared to the secondary structure predicted for the honeybee rRNAs [40], and the beginning and end of the genes were assumed to extend to the boundaries of the adjacent tRNA genes [41]. The A+T-rich region was checked for repeat motifs using REPFIND [42]. On the basis of the whole genome sequence and the annotation table exported from DOGMA, a GenBank file was created by using the Sequin 9.00 tool downloaded from GenBank. The file was imported into OrganellarGenomeDRAW for creating a graphical representation of the genome [43]. The complete mitochondrial genome sequence of *Philanthus triangulum* was deposited in the NCBI database (accession number JN871914).

### Phylogenetic analysis

All completed mitochondrial genomes of holometabolous insects available as of October 2010 (but only one species per genus) as well as the genomes of two hemimetabolous outgroup species (Hemiptera) were downloaded from the NCBI database (Table S3). The hymenopteran dataset was complemented with some unfinished genomes (*Primeuchroeus sp.*: nad2, nad3, nad5 missing; *Perga condei*: nad2 missing; *Nasonia vitripennis*: small part of nad2 missing).

Four nuclear genes were used for the comparative analysis of mitochondrial and nuclear substitution rates: the 18rRNA gene (18S), wingless (Wg; 336 bp), arginine kinase (ArgK; 1029 bp), and phosphoenolpyruvate carboxykinase (PEPCK; 732 bp). For 18S, an alignment based on the predicted secondary structure of the rRNA was obtained from the study of Whiting [44]. Four Apidae sequences were added manually to the alignment (*Anthophora montana* [AY995678], *Apis mellifera* [AB126807], *Centris rhodopus* [AY995680], and *Thyreus delumbatus* [AY995687]). For the three protein-coding genes, representative sequences from the major holometabolous insect orders Lepidoptera, Diptera, Hymenoptera and Coleoptera as well as outgroup specimens from the hemimetabolous Hemiptera were downloaded from the NCBI database (for accession numbers see Table S4). The ArgK sequence for *Philanthus triangulum* (JQ083477) was obtained by sequencing of a fragment that had been amplified by PCR with primers ArgK_fwd2 (5′-GACAGCAARTCTCTGCTGAAGAA-3′) and ArgK_KLTrev2 (5′-GATKCCATCRTDCATYTCCTTSACRGC-3′) [45].

For phylogenetic analyses, we aligned protein-coding sequences with ClustalX [46] and with T-Coffee [47] using the respective default parameters. To account for secondary structure of the rRNAs we used R-Coffee [48]. We checked the alignments by eye and corrected small portions of obvious homology missed by the alignment software (MacClade4 [49]) and selected final alignments on the basis of the fits of the alternatives as measured by the color-coded scoring system of T-Coffee and R-Coffee. All nuclear and mitochondrial gene alignments were submitted to TreeBASE.

For the combined analysis of all mitochondrial genes, the sequences were concatenated with MacClade4 [49]. We used likelihood tests in Phyml [50] to determine optimal models of sequence evolution from HKY, GTR, TN93 and their variants with invariant site (I) and rate heterogeneity (G). For the concatenated protein-coding genes, the Likelihood Ratio Test and the Bayesian Information Criterion (BIC) as implemented in Metapiga [51] were used to determine the optimal model. Based on these results, trees and parameters were optimized using MrBayes [52] assuming GTR+I+G maximum likelihood models partitioned by codon position for the protein-coding gene sequences. For the final analysis, the third codon position was excluded. Each analysis comprised two simultaneous runs with four chains each. The chains ran from 500,000 to 1,000,000 generations depending on when the average standard deviation of the split frequencies was consistently less than 0.01. Plots of the number of generations against the maximum likelihood scores indicated stabilization of likelihood scores. Further diagnostics included the potential scale reduction factor (PSRF) that measures the fit of branch length and all parameters. Trees and parameters from the first 25% of the generations were discarded (the burn in) after completion of the MCMC (Markov Chain Monte Carlo) search.

For the maximum likelihood analyses of the individual genes, we estimated parameters for GTR+I+G models of sequence evolution and optimized the tree using PHYML [50]. Our analysis of 1000 bootstrap replicates provided confidence limits on the maximum likelihood tree clades. For the concatenated sequences of several genes we used RAxML [53] to optimize the maximum likelihood tree and to calculate bootstrap proportions under GTR+I+G models partitioned by codon position.

Finally, we tested various alternative topologies (e.g. monophyly of the Hymenoptera and of the Diptera) using Shimodaira-Hasegawa [54] tests with GTR+I+G optimized parameters in PAUP*4b10 [55] to determine whether any differed significantly from the optimal tree. We inferred trees using the log-determinant transformation [56] to correct for base composition bias. We explicitly tested for base composition homogeneity across insect orders with an analysis of variance (ANOVA, JMP Pro 9.0.0) of the first two eigenvectors from a principal components analysis (PCA) of the base frequencies. Unlike the raw base frequencies, the PCA transformed variables (eigenvectors) are uncorrelated and approximately normally distributed and so amenable to ANOVA analysis.

We tested the sequences for the presence of a global molecular clock using the Rambaut multidimensional clock option in PAUP4b10 [55]. For computational facility, calculations of local clock parameters were calculated using maximum likelihood methods of Hyphy2.0 [57] on a smaller (50 taxa) data set rather than the full set. To test for significant differences in substitution rates across taxa for the various phylogenies (concatenated mitochondrial protein-coding genes, mitochondrial SSU and LSU rRNA, respectively, and the nuclear genes ArgK, PEPCK, wingless, and 18S rRNA), we used Hyphy's relative rates test. The Kishino80+G model was used for all datasets except for wingless, because relative rate parameters for this gene could not be resolved using the optimal model. Thus, we excluded the gamma parameter and used the suboptimal K80 test for the wingless dataset. For all analyses, substitution rates were compared for all possible pairwise combinations, using a hemipteran specimen as outgroup (except for the 18S rRNA dataset, for which *Libellula* [Odonata] was used as outgroup). Additional outgroup specimens were discarded from the analysis. Probability values were corrected for multiple comparisons with the Bonferroni correction.

## Results and Discussion

### Architecture of the beewolf mitochondrial genome

**Figure 1:**
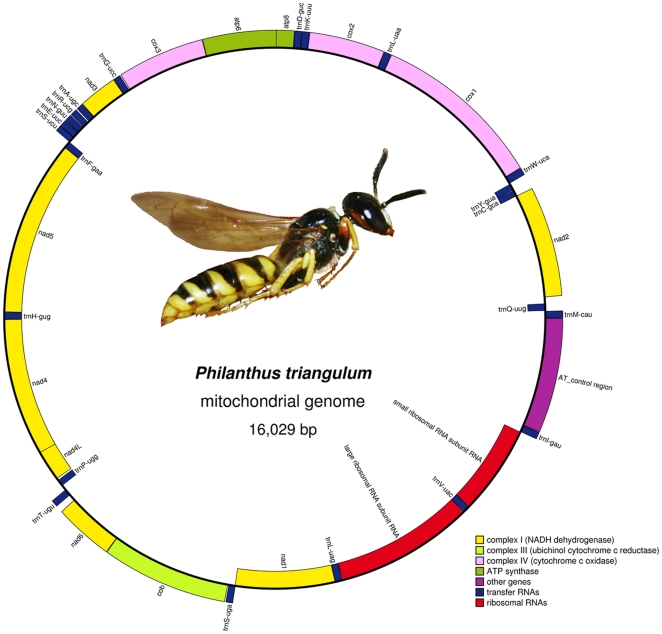
Mitochondrial genome sequence of *Philanthus triangulum*. Genes on the inside of the circle are located on the complementary strand (−). Genes are color-coded according to gene function: tRNA genes in blue, rRNA genes in red, genes of the NADH dehydrogenase complex in yellow, ATP synthase genes in dark green, cytochrome oxidase genes in light pink, cytochrome b in light green, and the AT-rich control region in magenta.

The mitochondrial genome of *P. triangulum* consists of 16,029 bp, with a mean A+T content of 83.6% (Fig. 1). The 37 genes typical for arthropod mitochondrial genomes were found (22 tRNA genes, two rRNA genes, and 13 protein-coding genes; Table S5). While the protein-coding and rRNA genes were conserved in positions and orientations relative to the inferred ancestral arrangement in insect mitochondrial genomes [58], five translocations of tRNAs were detected (tRNA-Met/-Gln; tRNA-Trp; tRNA-Glu/-Ser(AGN); tRNA-Pro/-Thr; tRNA-Ile). The organization of the highly variable nad3-nad5 junction was identical to that of *Vespula germanica* and differed only in the relative order of tRNA-Glu/-Ser(AGN) from the inferred ancestral type in insects [59].

### Coding density

As is characteristic for mitochondrial genomes in general, the mt-genome of *P. triangulum* exhibits an extremely high coding density. Overlaps between protein-coding regions and/or tRNA genes were found in six locations, with a total of 26 bases shared by two genes (Table S5). tRNA genes in animal mitochondrial genomes have commonly been found to overlap at the discriminator nucleotide [60], and shared nucleotides between protein coding genes and tRNA genes have also been reported for many insect species [11], [41], [61]–[64]. If the A+T-rich region and potential non-coding regions between the rRNA genes and adjacent tRNA genes are not taken into account, a total of 217 bp of non-coding intergenic spacers was found (size range of non-coding regions: 1–61 bp). Thus, coding density in the mitochondrial genome of *P. triangulum* tends to be even higher than in other hymenopteran mt-genomes (e.g. 811 bp non-coding sequences in *Apis mellifera* [61]; 486 bp in *Melipona bicolor* [41]; 880 bp in *Bombus ignitus* [64]), but slightly lower than in *Drosophila yakuba* (183 bp [62]).

### Protein-coding genes

The 13 protein-coding genes typical for animal mitochondrial genomes were found in the mt-genome of *P. triangulum* (Fig. 1, Table S5). Conventional start codons ATG or ATT could be assigned to all protein-coding genes except for nad3 and cox2 (Table S5). The nad3 gene apparently uses ATC (codes for Ile) as a start codon, while the start codon for cox2 seems to be TTG (codes for Leu). The use of irregular start codons has been found repeatedly for animal mitochondrial genomes (notably for cox1, e.g. [60]–[63], [65]), but to our knowledge this is the first report of a TTG start codon in cox2 (but GTG has recently been found as the start codon for cox2 in *Eriogyna pyretorum*, Lepidoptera: Saturniidae, see [66]). Six of the protein-coding genes end in complete stop codons (TAA). The remaining seven protein-coding genes appear to use abbreviated stop codons (TA or T) that are presumably completed by post-transcriptional polyadenylation as reported for other animal mitochondrial genomes [67]–[69]. The nucleotide composition of the *P. triangulum* mt-genome was strongly biased towards A+T, with a mean A+T content of 83.6%, which lies in the range found for other hymenopteran mt-genomes (Table S6) [9], [41], [61], [63], [64], [70]. Within protein-coding genes, the nucleotide bias differed strongly among codon positions. The highest A+T bias was found for the third positions (94.7%), while the first and second positions had A+T contents lower than the average of the complete genome (79.0% and 74.4%, respectively) (Table S6).

### Ribosomal and transfer RNAs

The anticodons of the tRNAs in the European beewolf mitochondrial genome were identical to those of *Bombus ignitus* [64] and *Apis mellifera* [61], but differed from those found for other insects: in *P. triangulum*, GAT is the anticodon of tRNA-Ile, TTT of tRNA-Lys, and TCT of tRNA-Ser(AGN), while these are CCT, CTT, and GCT in most other insects, respectively [64]. Of the 22 tRNAs, 21 showed the typical cloverleaf secondary structure. As in several other insect species, the tRNA-Ser(AGN) showed an aberrant structure, with the D-arm forming a simple loop [64], [71].

Two rRNAs were found in the mitochondrial genome of *P. triangulum*: rrnL is located between tRNA-Leu(CTN) and tRNA-Val, and rrnS between tRNA-Val and tRNA-Ile. Their lengths (1328 bp for rrnL and 863 bp for rrnS) are similar to those of other hymenopteran mt-rRNAs [61], [64].

### A+T-rich region

Both length (1039 bp) and AT-content (85.7%) of the A+T-rich region in the beewolf mt-genome are within the range of other insect mitochondrial genomes (Table S6) [64], [72]. This region is by far the longest non-coding sequence in the beewolf mitochondrial genome, and it is likely to play a role in the initiation of replication as well as the regulation of transcription, as has been shown for *Drosophila yakuba* [62], [73]. In the A+T-rich region of the beewolf mitochondrial genome, a 43 bp-tandem repeat (two repeat copies) as well as several (AT)_n_ microsatellite-like tandem repeats of up to 13 copies were discovered. Such repeats are typical for insect mitochondrial A+T-rich regions and may cause heteroplasmy [72].

### Phylogeny of holometabolous insects based on mitochondrial genomes

**Figure 2:**
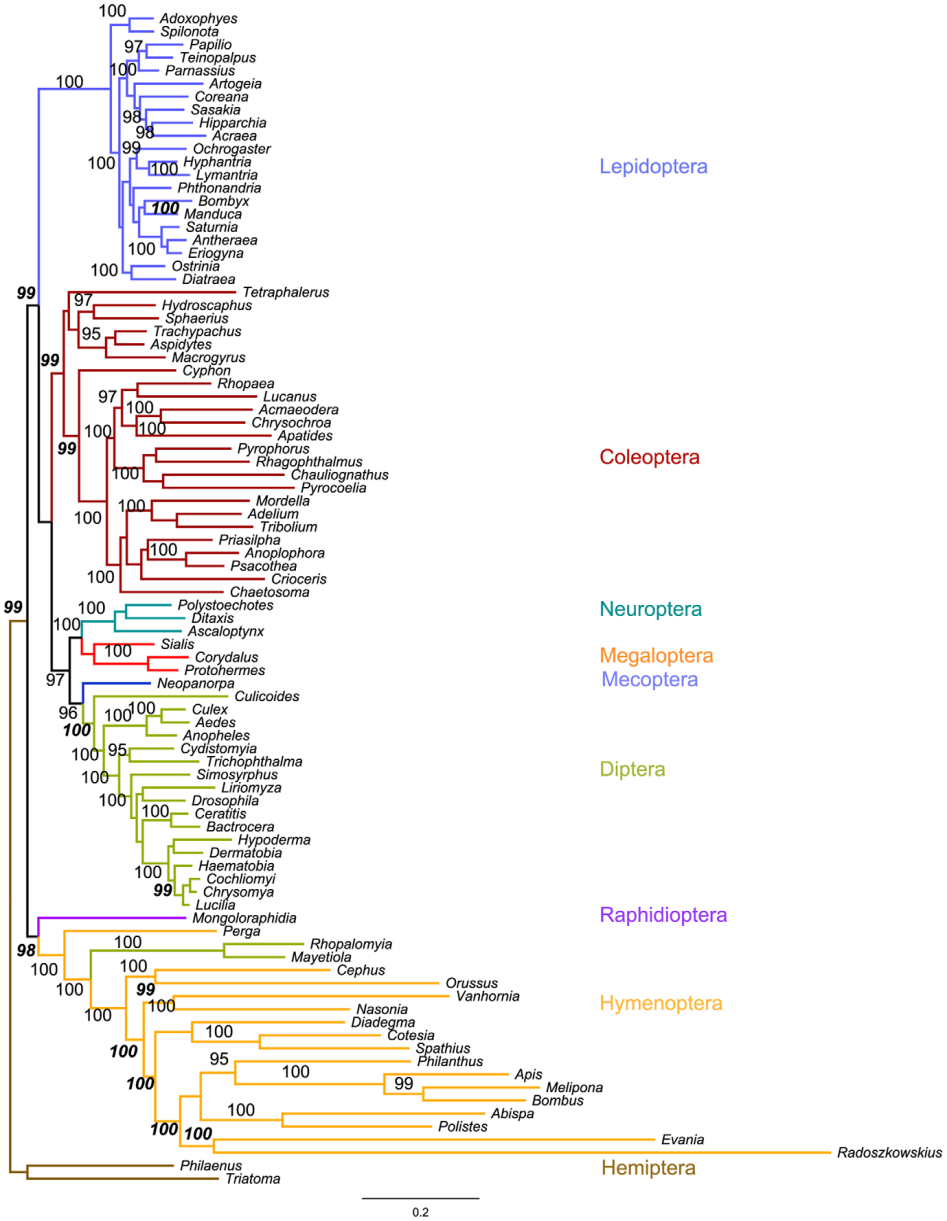
Bayesian phylogeny inferred from the 1^st^ and 2^nd^ codon positions of the 13 mitochondrial protein-coding genes. Because most of the maximum likelihood values differed very little from the posterior probabilities, branches are labeled with a single number for reading clarity. Most are maximum likelihood bootstrap proportions (regular text) but are replaced by posterior probabilities (bold italics) for branches with a difference of more than five between the two values. Branches are color-coded based on order-level taxonomic affiliations.

**Figure 3:**
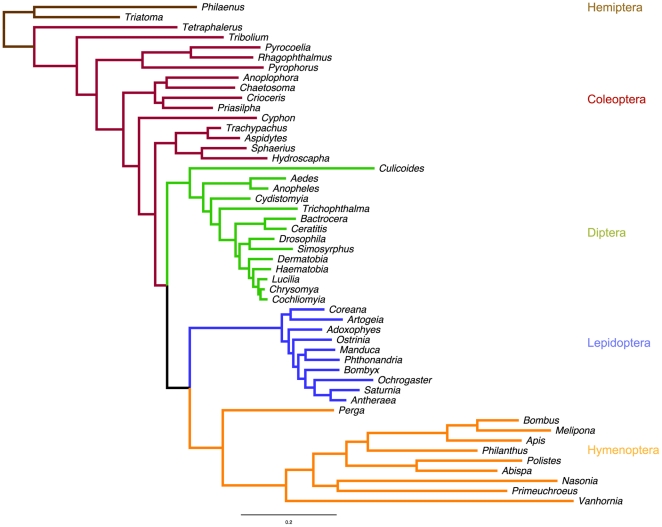
Maximum likelihood tree inferred from the mitochondrial 12S and 16SrRNA gene sequences of 50 representative taxa from the four major holometabolous insect orders. Branches are color-coded based on order-level taxonomic affiliations (see Fig. 2).

Bayesian and maximum likelihood analyses were used to reconstruct the phylogeny of holometabolous insects based on mitochondrial protein-coding and rRNA genes. Analyses including the 1^st^ and 2^nd^ positions of all protein-coding genes recovered most of the expected relationships on the order, suborder, superfamily and family level, with three notable exceptions: The gall midge genera *Rhopalomyia* and *Mayetiola* (Diptera, Cecidomyiidae) were consistently misplaced in the Hymenoptera; the only Raphidiopteran was placed at the base of the Hymenoptera instead of within the Neuropterida (Neuroptera+Mecoptera); and the non-aculeate genus *Evania* erroneously grouped as a sister group of *Radoszkowskius* within the Aculeata (Fig. 2) [74], [75]. As in previous phylogenetic studies, the Hymenoptera were recovered as the most basal of the holometabolous insect orders [75], [76], Diptera and Mecoptera were sister groups and together represented the sister group of the Neuropterida [77], which disagrees with a recent study based on single-copy nuclear genes that revealed the Coleoptera and Strepsiptera to be the closest relatives to the Neuropterida [75]. Including the 3^rd^ positions in the phylogenetic analysis led to the loss of monophyly in the Coleoptera, and an analysis with only the 3^rd^ positions did not recover the Coleoptera or the Lepidoptera as monophyletic clades, and several of the expected relationships below order level were not recovered as monophyletic clades (Fig. S1). A maximum likelihood phylogeny of the two mitochondrial rRNA genes with a subset of 50 taxa recovered the Lepidoptera, Diptera, and Hymenoptera as monophyletic, but not the Coleoptera or many of the relationships on lower taxonomic levels (Fig. 3). Our results agree with an earlier study indicating that Bayesian analyses of 1^st^ and 2^nd^ protein-coding positions are most useful to recover uncontroversial relationships within the Hymenoptera when using mitochondrial genomes [8].

**Figure 4:**
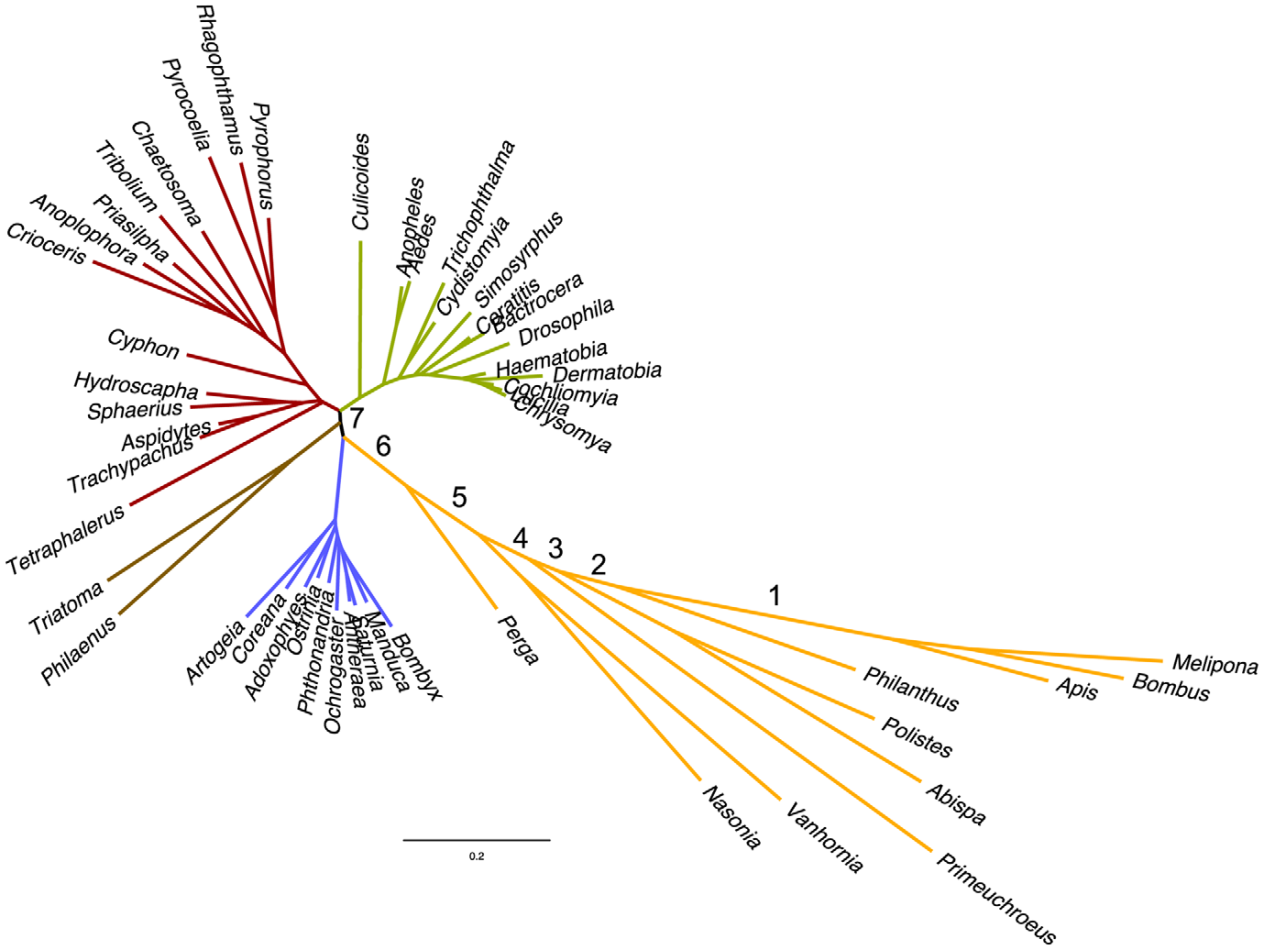
Molecular clock analysis of 50 representative taxa from the four major holometabolous insect orders (global molecular clock lnL = −311050, no clock lnL = −309424). Numbers correspond to significantly different local clock log likelihood values as follows: ΔlnL = 24 df = 2 (branch 1), ΔlnL = 125 df = 3(branch 2), ΔlnL = 170 df = 5 (branch 3), ΔlnL = 172 df = 6 (branch 4), ΔlnL = 263 df = 8 (branch 5), ΔlnL = 817 df = 9 (branch 6), ΔlnL = 2363 df = 19 (branch 7). Branches are color-coded based on order-level taxonomic affiliations (see Fig. 2).

All phylogenetic analyses showed markedly longer branches for the apocritan Hymenoptera as well as for *Orussus* and, to a lesser extent, *Cephus*, than for any other taxa (with the exception of the two dipteran genera *Rhopalomyia* and *Mayetiola*, which will be discussed below), indicating unusually high mitochondrial substitution rates in the Hymenoptera. High substitution rates in hymenopteran mt-genes have been reported repeatedly and implicated in the problems of reconstructing holometabolous insect phylogenies based on mitochondrial genes due to long-branch attraction effects [8], [11], [18]. Although tree topologies varied markedly among single genes, we observed long branches leading to hymenopteran taxa for all protein-coding genes with the possible exception of Atp8 (see Fig. S2). Molecular clock analyses including all protein-coding genes showed that the substitution rates within Hymenoptera deviated significantly from molecular clock assumptions (Fig. 4). Relative rate tests confirmed the significant differences between Hymenoptera and the other major holometabolous insect orders for the concatenated protein-coding gene dataset and to a lesser extent for the SSU rRNA, but not for the LSU rRNA (Table 1).

**Table 1:** Comparative analysis of relative substitution rates in hymenopteran and non-hymenopteran taxa for three mitochondrial and four nuclear phylogenies.

			Number of tests			
Genome	Genes	Pairwise comparisons	signif.(1)	non-signif.	total	% signif.
**Mito**	**all protein-**	**Apocrita vs. non-Hymenoptera**	**930**	**6** (2)	**936**	**99**
	**coding**	**Apocrita vs. Tenthredinoidea (** ***Perga*** **)**	**13**		**13**	**100**
	**genes**	**Apocrita vs. Cephoidea (** ***Cephus*** **)**	**12**	**1**	**13**	**92**
	**combined**	**Apocrita vs. Orussoidea (** ***Orussus*** **)**	**7**	**6**	**13**	**54**
		Apocrita vs. Apocrita	29	49	78	37
		**Orussoidea (** ***Orussus*** **) vs. non-Hymenoptera**	**70**	**2** (3)	**72**	**97**
		**Orussoidea (** ***Orussus*** **) vs. Tenthred. (** ***Perga*** **)**	**1**		**1**	**100**
		Orussoidea (*Orussus*) vs. Cephoidea (*Cephus*)		1	1	0
		**Cephoidea (** ***Cephus*** **) vs. non-Hymenoptera**	**69**	**3** (4)	**72**	**96**
		Cephoidea (*Cephus*) vs. Tenthred. (*Perga*)		1	1	0
		Tenthredinoidea (*Perga*) vs. non-Hymenoptera	22	50	72	31
		non-Hymenoptera vs. non-Hymenoptera	239(5)	2317	2556	9
		*Total*	*1392*	*2436*	*3828*	*36*
	**SSU rRNA**	**Apocrita vs. non-Hymenoptera**	**177**	**165**	**342**	**52**
		Apocrita vs. Symphyta (Perga)		9	9	0
		Apocrita vs. Apocrita		36	36	0
		Symphyta (Perga) vs. non-Hymenoptera	2	36	38	5
		non-Hymenoptera vs. non-Hymenoptera	3	700	703	0
		*Total*	*182*	*946*	*1128*	*16*
	**LSU rRNA**	Apocrita vs. non-Hymenoptera	17	325	342	5
		Apocrita vs. Symphyta (Perga)		9	9	0
		Apocrita vs. Apocrita		36	36	0
		Symphyta (Perga) vs. non-Hymenoptera		38	38	0
		non-Hymenoptera vs. non-Hymenoptera	4	699	703	1
		*Total*	*21*	*1107*	*1128*	*2*
**Nuclear**	**ArgK**	Hymenoptera vs. non-Hymenoptera		98	98	0
		Hymenoptera vs. Hymenoptera		21	21	0
		non-Hymenoptera vs. non-Hymenoptera		91	91	0
		*Total*	*0*	*210*	*210*	*0*
	**PEPCK**	Hymenoptera vs. non-Hymenoptera		350	350	0
		Hymenoptera vs. Hymenoptera	1	90	91	1
		non-Hymenoptera vs. non-Hymenoptera		300	300	0
		*Total*	*1*	*740*	*741*	*0*
	**wingless**	Hymenoptera vs. non-Hymenoptera	42	322	364	12
		Hymenoptera vs. Hymenoptera	9	69	78	12
		non-Hymenoptera vs. non-Hymenoptera		378	378	0
		*Total*	*51*	*769*	*820*	*6*
	**18S rRNA**	**Hymenoptera vs. non-Hymenoptera**	**506**	**454**	**960**	**53**
		Hymenoptera vs. Hymenoptera		190	190	0
		**non-Hymenoptera vs. non-Hymenoptera**	**564**	**564**	**1128**	**50**
		*Total*	*1070*	*1208*	*2278*	*47*

(1) significant at *P*<0.01 after Bonferroni correction.

(2) all involving *Rhopalomyia* or *Mayetiola.*

(3) *Orussus* vs. *Rhopalomyia* and *Mayetiola.*

(4) *Cephus* vs. *Rhopalomyia*, *Mayetiola*, and *Pyrocoelia.*

(5) of those 140 involving *Rhopalomyia* or *Mayetiola.*

Given are the numbers of significant (after Bonferroni-correction) and non-significant pairwise comparisons of relative rates, as well as the proportion of significant tests (in percent). Comparisons with >50% significant tests are highlighted in bold. To elucidate the origin of elevated substitution rates in Hymenoptera, the results for the basal symphytan taxa are listed individually for the genes for which sequences of these taxa were available. A hemimetabolous species was used as the outgroup for each analysis.

Surprisingly, more detailed analyses of the basal Hymenoptera (“Symphyta”) indicate that elevated substitution rates are not confined to the Apocrita, as had been suggested earlier [18], but also occur in *Orussus* (Orussoidea) and *Cephus* (Cephoidea), both of which show significantly higher rates than >95% of all investigated non-hymenopteran taxa (with 2/2 and 2/3 non-significant pairwise comparisons involving the dipteran taxa *Rhopalomyia* and *Mayetiola*, respectively, which also exhibit unusually high substitution rates [see below]). Even in *Perga condei* (Tenthredinoidea), more than 30% of the pairwise comparisons suggest elevated substitution rates as compared to non-hymenopteran taxa, which is significantly more than the proportion of significant paiwise tests among non-hymenopterans (9.35%, Chi^2^ = 35.2, *P*<0.001). It has to be noted, however, that all three symphytan taxa show significantly lower substitution rates than most Apocrita (Table 1), so their substitution rates should be considered intermediate between Apocrita and the non-hymenopteran taxa.

**Figure 5:**
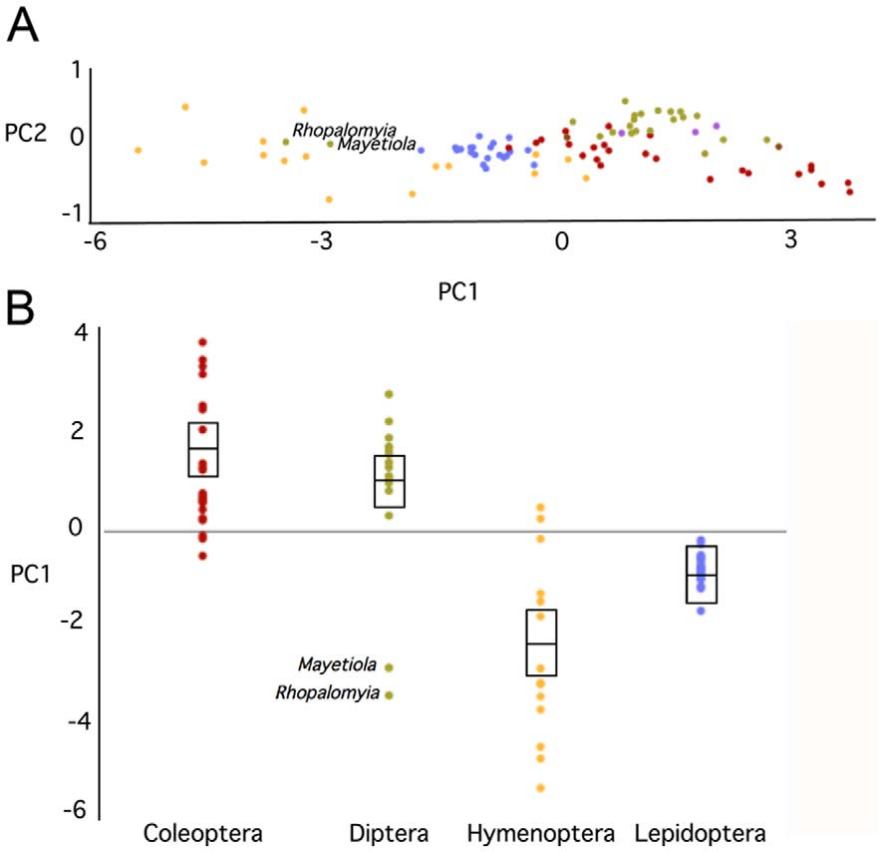
Principal components analysis of base frequencies among mitochondrial genome sequences. **A** Plot of the first principal component against the second principal component (explaining 93% and 5% of the variability, respectively). **B** Boxplot of PC1 according to insect orders. An ANOVA of the first principal component shows that the base composition in Hymenoptera is significantly biased relative to the other orders (*P*<0.01 for all pairwise comparisons involving Hymenoptera), due to very high AT frequencies. Samples are color-coded based on order-level taxonomic affiliations (see Fig. 2).

Our analyses consistently misplaced the gall midge genera *Rhopalomyia* and *Mayetiola* within the Hymenoptera rather than the Diptera (Fig. 2), and a Shimodaira-Hasegawa test significantly rejected a tree with a monophyletic dipteran clade (ΔlnL = 733.3, *P*<0.0001). To explain the presumably false attraction of these dipteran sequences to the hymenopteran clade, we investigated sources of bias uncorrected by the optimal GTR+I+G model. First, we used a logDet transform [56] that mitigates the effect of base composition bias among the sequences. This tree, however, also included the two dipteran sequences in the hymenopteran clade (data not shown). In order to statistically test for homogeneity of base composition across the sequences, we transformed the four correlated base frequency variables to uncorrelated, normally distributed variables with principal component analysis (Table S7). The resulting first two principal components (PCs) explain 93% and 5% of the variance, respectively, and the sign and magnitude of the eigenvectors indicates that the first PC contrasts AT against CG frequencies across the sequences. A scatterplot of the two PCs shows that many of the Hymenoptera and the two Diptera (*Rhopalomyia and Mayetiola*) cluster in a group separated from most of the other sequences (Fig. 5A). A comparison of PC1 across insect orders confirmed that the Hymenoptera differ significantly in base composition from all of the other three major holometabolous insect orders (Fig. 5B; ANOVA, *P*<0.001; Tukey HSD post-hoc tests: *P*<0.01 for all pairwise comparisons including Hymenoptera). However, both *Rhopalomyia* and *Mayetiola* exhibit base composition biases that are in the range of Hymenoptera rather than other Diptera. Thus, the misplacement of these gall midge genera within the Hymenoptera is likely due to their bias in base composition (especially the extreme AT bias) that is more similar to the Hymenoptera than to the Diptera. Although the authors of the original description of the *Rhopalomyia* and *Mayetiola* mitochondrial genomes did not report on elevated substitution rates in gall midges as compared to other Diptera, they commented on several unusual features like the very small size, the rearrangements and truncation of tRNA genes, and, notably, an unusually high AT content in the coding regions [78].

### Nuclear phylogeny of holometabolous insects

**Figure 6:**
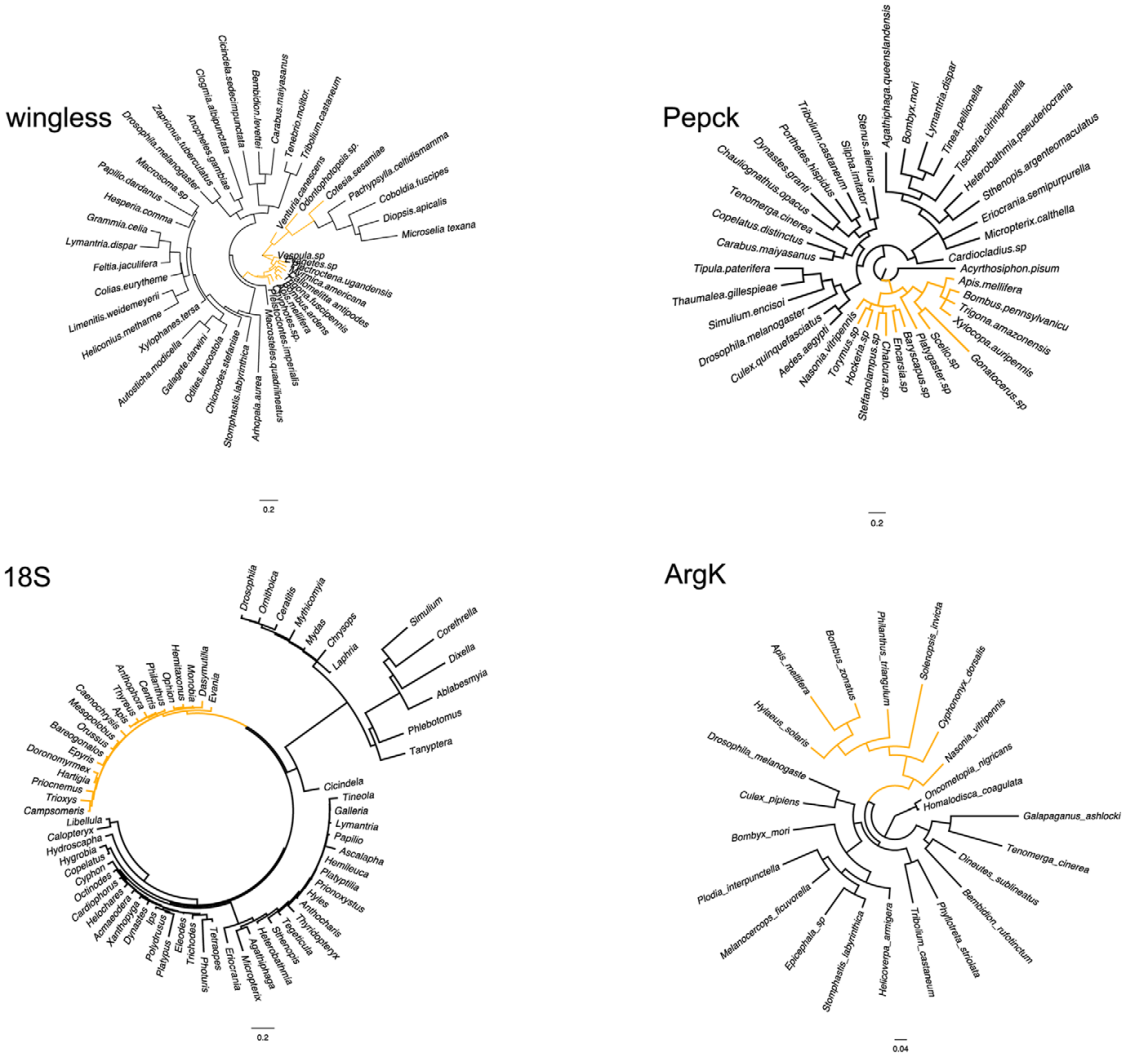
Phylogenetic trees from analyses of four nuclear genes for representative taxa of the four major holometabolous insect orders. Sequences for the analysis of wingless, PEPCK, and ArgK were obtained from the NCBI database, the 18S dataset represented a reduced dataset from the analysis of Whiting (2002) that was supplemented with some additional taxa of Apidae from the NCBI database. Hymenopteran taxa are highlighted by yellow branches.

Based on four nuclear gene datasets, we reconstructed the phylogenetic relationships among holometabolous insect orders (Fig. 6). While PEPCK and ArgK recovered the four major holometabolous insect orders as monophyletic, wingless failed to group the hymenopteran taxa in a monophyletic clade, and the Coleoptera were paraphyletic in the 18S phylogeny, as had been reported earlier [44]. Relative rate tests yielded no evidence for higher substitution rates in Hymenoptera for the any of the four nuclear genes (Table 1). For both wingless and 18S rRNA, the observed differences in relative rates between hymenopteran and non-hymenopteran taxa were due to lower rather than higher rates in Hymenoptera (all 42 and 506 significant differences for wingless and 18S rRNA, respectively). These results are in contrast to an earlier study suggesting that both mitochondrial and nuclear genes exhibit elevated substitution rates [21]. However, other phylogenetic analyses of the extant hexapod orders based on nuclear genes did not comment on conspicuously elevated substitution rates in Hymenoptera as compared to other hexapod lineages (see [44], [79], but Whiting et al. [80] comment briefly on long branches in Hymenoptera) or found even shorter phylogenetic branches leading to Hymenoptera than other insect orders [76], which agrees with our results for wingless and 18S rRNA.

### Comparison of mitochondrial and nuclear phylogenies

Our results indicate that substitution rates in Hymenoptera as compared to other holometabolous insect orders are significantly and consistently elevated for mitochondrial (with the exception of the LSU rRNA) but not for any of the four nuclear genes under investigation (Table 1). Interestingly, the inclusion of several non-apocritan Hymenoptera (*Perga, Cephus, Orussus*) provides evidence for elevated mitochondrial substitution rates also in *Orussus*, to a lesser extent in *Cephus*, and possibly even in *Perga*. Thus, either substitution rates of mitochondrial genes already started to increase within the paraphyletic suborder Symphyta prior to the origin of the Apocrita, or the elevated rates evolved independently in the Apocrita and in the only ectoparasitic group within the Symphyta, the Orussoidea, as has been suggested previously by Dowton and Austin [18]. However, the intermediate to high branch lengths and the significantly elevated substitution rates found for *Cephus* in our analyses provide support for the former hypothesis (see Table 1, Fig. 2 and S1).

Mechanistically, substitution rates are determined by the mutation rate and the probability that a given mutation reaches fixation [81]. Although analyses based on sequence comparisons fail to record mutations that never reach fixation and generally do not distinguish between the effects of mutation rate and probability of fixation, it is important to consider these as two distinct evolutionary factors affecting substitution rates [82]. Mutation rates are generally thought to be positively correlated with metabolic rate and mutational pressure (e.g. mutagens, oxidative stress), but negatively with the efficiency of the DNA repair machinery. Fixation rates, on the other hand, depend on the effective population size, cladogenesis rate, and the selective pressures acting upon the gene of interest [83]. In many of these factors, mitochondrial and nuclear genomes differ markedly, which may explain the generally higher substitution rates observed in mitochondrial as compared to nuclear genomes [84], [85]. Additionally, Oliveira et al. [70] proposed the compensation-draft-feedback model to explain the high substitution rates in *Nasonia spp.* mitochondrial genomes: Due to the lack of recombination in mitochondria, any fixation of an adaptive mitochondrial mutation can drag along mildly deleterious substitutions in the same haplotype. Compensatory mutations in the same or in interacting mitochondrial genes would then be selected for, which can lead to a second selective sweep to fixation. Thus, this mechanism could potentially result in a cascade of adaptive and non-adaptive mutations in mitochondrial but not nuclear genes, because recombination impedes the compensation-draft-feedback mechanism [70]. However, the available mechanistic models fail to explain the exceptionally high substitution rates observed in mitochondrial genomes of Hymenoptera as compared to other holometabolous insect orders.

Previously, an ecological hypothesis has been proposed to account for the high mt-substitution rates in Hymenoptera as well as in lice (Phthiraptera) [15], [16] and mites [19]: In all three taxa, ectoparasitism is deemed to be the current (lice and mites) or ancestral (apocritan Hymenoptera) lifestyle [86], [87], which entails short generation times and frequent founder events with small effective population sizes. Since these factors are known to be associated with increased substitution rates, the parasitic lifestyle has been hypothesized to be responsible for the elevated evolutionary rates in these insect lineages [16], [18], [19], [21]. *A priori*, however, the hypothesis predicts elevated substitution rates in both mitochondrial and nuclear genes, and we fail to find evidence for the latter in our nuclear gene phylogenies of Hymenoptera. In addition, it has been noted earlier that several parasitic lineages do not show elevated mitochondrial substitution rates or higher numbers of gene rearrangements [21], [88], [89]. Furthermore, our analyses suggest that mitochondrial substitution rates already began to increase in a non-parasitic ancestor before the origin of the Apocrita and continue to do so in the extant apocritan families, despite the fact that several of them are not parasitic anymore (Fig. 2).

In conclusion, our results do not provide support for the hypothesis that the parasitic lifestyle alone can explain the pattern of evolutionary rates observed in insect mitochondrial genomes. Considering the immense ecological diversity of Hymenoptera, it seems possible that elevated mitochondrial mutation rates evolved originally as a pleiotropic or epistatic side-effect of an adaptive (mitochondrial or nuclear) mutation. Subsequent processes like the compensation-draft feedback may have led to an increase in fixation rates and a further accumulation of mutations, thus resulting in the strongly elevated substitution rates in Hymenoptera that we observe today.

## Supporting Information

Figure S1 **Bayesian phylogeny inferred from the 3^rd^ codon postions of 13 mitochondrial protein-coding genes.** Branches are color-coded based on order-level taxonomic affiliations (see Fig. 3).
(TIF)

Figure S2 **Bayesian phylogenies inferred from single mitochondrial protein-coding genes.**
(TIF)

Table S1 Primers used for amplification and sequencing of the mitochondrial genome of *P. triangulum*.
(DOCX)

Table S2 Primer combinations and PCR conditions used for the amplification of the mitochondrial genome of *P. triangulum*.
(DOCX)

Table S3 Taxonomy and GenBank accession numbers of mitochondrial genomes used for the phylogenetic analysis.
(DOCX)

Table S4 Taxonomy and GenBank accession numbers of nuclear genes used for the phylogenetic analysis.
(DOCX)

Table S5 Summary of the mitochondrial genome of *P. triangulum*.
(DOCX)

Table S6 Nucleotide composition of the protein-coding, ribosomal RNA and transfer RNA genes in the mitochondrial genome of *P. triangulum*. Nucleotide frequencies for all genes were calculated for the coding strand. The strand coding for the cox1-3 genes (+) was arbitrarily chosen for the calculation of the average nucleotide frequencies of the complete genome (“All sites”).
(DOCX)

Table S7 Principle Components Analysis (PCA) of base composition in mitochondrial genomes of holometabolous insects. The 1st principle component accounts for most of the variability (93%) among the four nucleotides and contrasts A and T frequencies against C and G frequencies.
(DOCX)
