# Supplementary figures and images for: Accelerated Evolution of Mitochondrial but Not Nuclear Genomes of Hymenoptera: New Evidence from Crabronid Wasps
Source: PLoS One. 2012 Mar 6;7(3):e32826. doi: 10.1371/journal.pone.0032826 (PMC3295772; doi:10.1371/journal.pone.0032826)

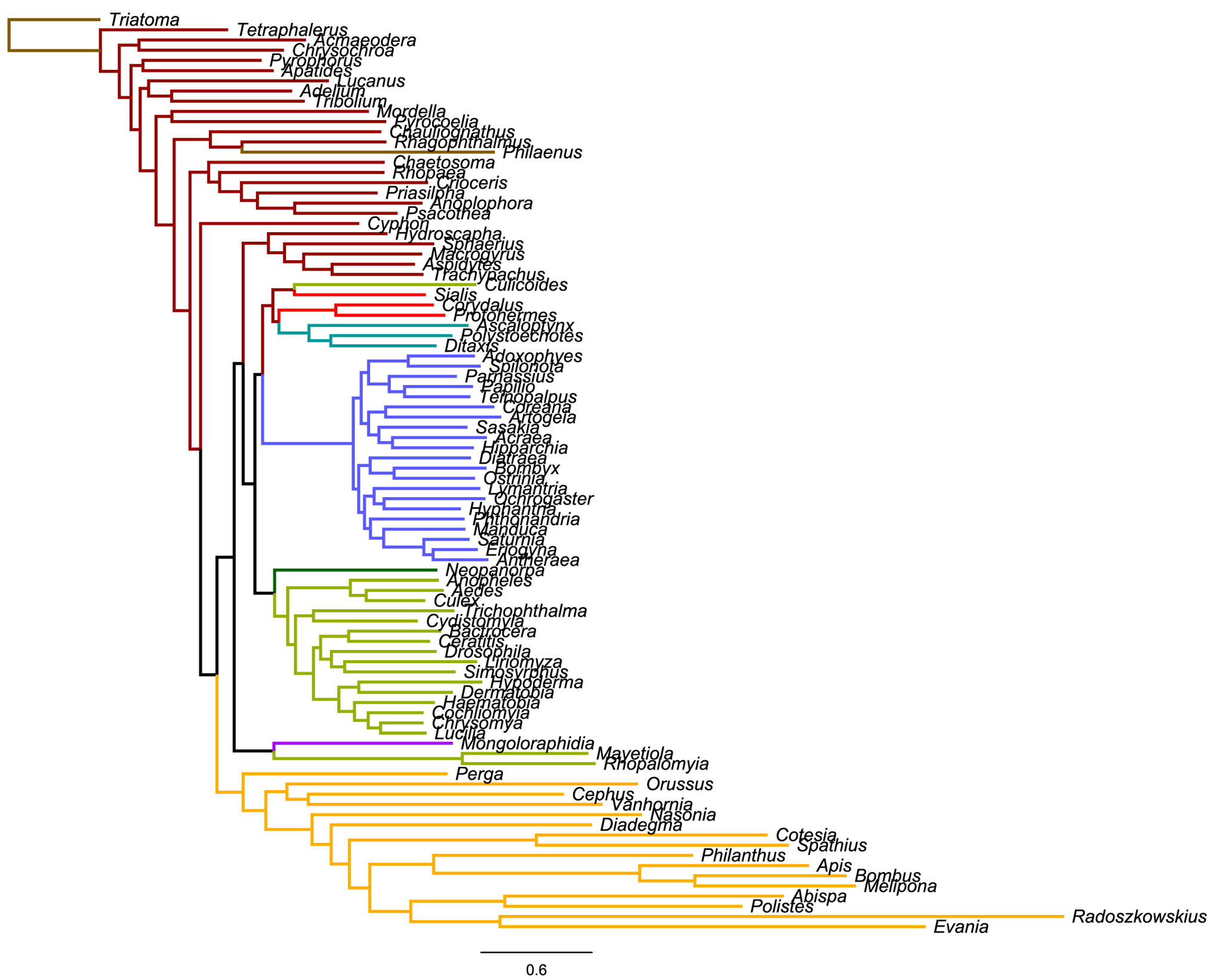

Supplement: Figure S1 — Bayesian phylogeny inferred from the 3rd codon postions of 13 mitochondrial protein-coding genes. Branches are color-coded based on order-level taxonomic affiliations (see Fig. 3). (TIF) [file pone.0032826.s001.tif]

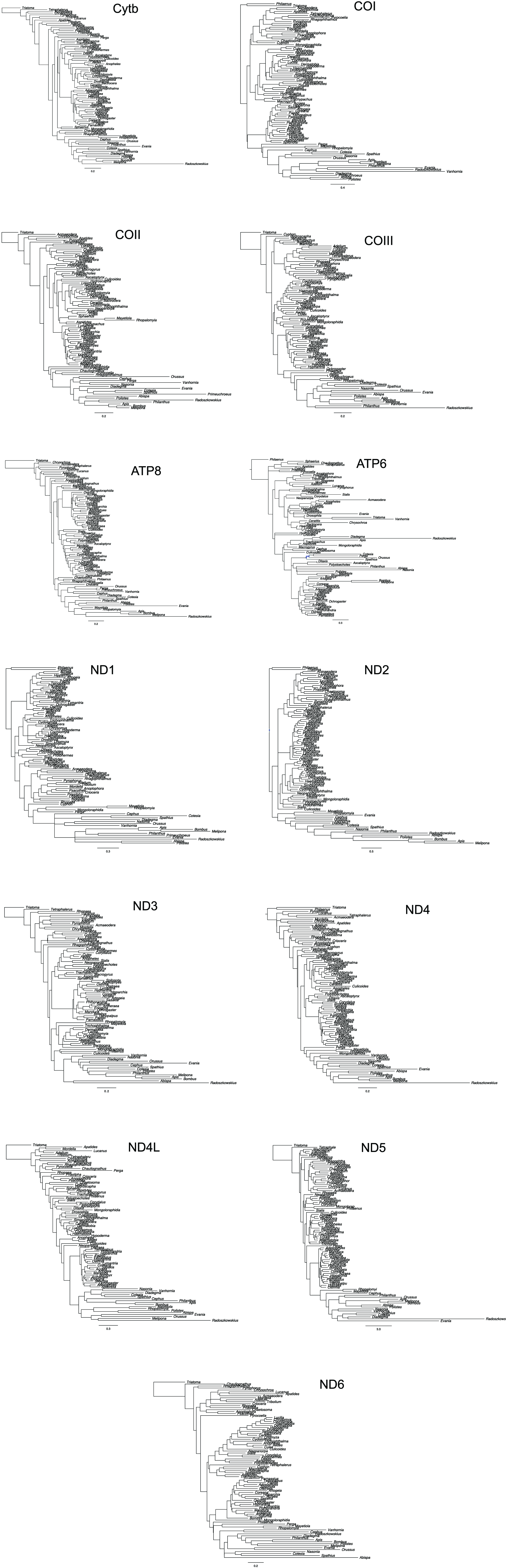

Supplement: Figure S2 — Bayesian phylogenies inferred from single mitochondrial protein-coding genes. (TIF) [file pone.0032826.s002.tif]
