# Supplementary material for: Accelerated Evolution of Mitochondrial but Not Nuclear Genomes of Hymenoptera: New Evidence from Crabronid Wasps
Source: PLoS One. 2012 Mar 6;7(3):e32826. doi: 10.1371/journal.pone.0032826 (PMC3295772; doi:10.1371/journal.pone.0032826)
Supplement: Table S1 — Primers used for amplification and sequencing of the mitochondrial genome of P. triangulum. (DOCX) [file pone.0032826.s003.docx]

**Table S1**: Primers used for amplification and sequencing of the mitochondrial genome of *P. triangulum*.

|  |  |  |  |  |  |
| --- | --- | --- | --- | --- | --- |
|  |  | ***P. triangulum*** |  |  |  |
| **Primer** | **Region** | **position** | **fwd/rev** | **5' - 3' sequence** | **Reference** |
| ATRR2 | atr | 116-140 | rev | ATTATATTTTGGTGTATTAAGCACT | this study |
| N2LR2 | nad2 | 372-397 | rev | TGGATGAAATTGTTTGAAAAATAAAG | this study |
| N2LR1 | nad2 | 490-513 | rev | TCATGAATGAAATGGTATAATTCC | this study |
| N2RR1 | nad2 | 953-977 | rev | TTAATTAATTGTTCTGTTGCTGATC | this study |
| LCO | cox1 | 1419-1443 | fwd | GGTCAACAAATCATAAAGATATTGG | Folmer et al. 1994 |
| C1LR2 | cox1 | 1767-1787 | rev | GGGGGTAAAGTGTTCAACCAG | this study |
| C1LR1 | cox1 | 1991-2010 | rev | GCTCCAGCAAGAACTGGAAG | this study |
| Jerry | cox1 | 2090-2112 | fwd | CAACATTTATTTTGATTTTTTGG | Simon et al. 1994 |
| Ben | cox1 | 2504-2526 | rev | GCWACWACRTAATAKGTATCATG | Kronauer et al. 2004 |
| C2LR1 | cox2 | 3034-3057 | rev | TTAATGATCCTGAGTATTGTAAAG | this study |
| C2LF2 | cox2 | 3168-3195 | fwd | CGAAACTTATTACATGGAAATACAATTG | this study |
| C2LF1 | cox2 | 3308-3334 | fwd | AGTAATTGGTCATCAGTGATATTGATC | this study |
| Barbara_mod | cox2 | 3591-3614 | rev | TCCACAAATTTCTGAACATTGTCC | this study |
| A8LF1 | atp8 | 3829-3848 | fwd | TTAATCCCTCAGACAAGACC | this study |
| C3MF1 | cox3 | 5085-5105 | fwd | TTTCACTAACATGAGCACACC | this study |
| C3RF1 | cox3 | 5259-5279 | fwd | GAACTGGATTTCACGGTCTTC | this study |
| C3RR1 | cox3 | 5385-5409 | rev | AATCAAATTACGTCTACAAAATGTC | this study |
| TARF1 | nad3-nad5 | 6015-6038 | fwd | AATTAATTGAAACCAAAATAGAGG | this study |
| N5MR2 | nad5 | 7328-7349 | rev | CTGCTATAGCTGCTCCTACTCC | this study |
| N5RF1 | nad5 | 7587-7614 | fwd | GACAATAAGAAATTAATCCTAAACCATC | this study |
| N5RR1 | nad5 | 7716-7738 | rev | TAGTGTAGATTATATATCTGAAG | this study |
| N4LF1 | nad4 | 8278-8301 | fwd | AAGGAGCTGCTATATTAGAAGAAC | this study |
| ND4 | nad4 | 8712-8731 | fwd | GGAGCTTCAACATGAGCTTT | Simon et al. 1994 |
| N4RF1 | nad4 | 9406-9426 | fwd | AGCCAATCCTATAATTCTTTC | this study |
| CB1 | cob | 10825-10850 | fwd | TATGTACTACCATGAGGACAAATATC | Simon et al. 1994 |
| CBLR5 | cob | 11035-11058 | rev | TGGATTTCTAGATCCAGTTCTGTG | this study |
| CBRF1 | cob | 11456-11479 | fwd | AAGAAATTGAGTACCCTTTCATCC | this study |
| N1RR1 | nad1 | 12463-12482 | rev | ATTCAAGATCGAAAAGGTCC | this study |
| 16Sbr | rrnL | 12776-12797 | fwd | CCGGTCTGAACTCAGATCACGT | Simon et al. 1994 |
| LRLR1 | rrnL | 12900-12923 | rev | GGGATAACAGCGTTATATCTTTGG | this study |
| LRLF2 | rrnL | 12905-12928 | fwd | GATATAACGCTGTTATCCCTAAGG | this study |
| 16Sar | rrnL | 13314-13333 | rev | CGCCTGTTTAACAAAAACAT | Simon et al. 1994 |
| LRRF1 | rrnL | 13696-13718 | fwd | ACGAATAACATTTCATTCCTAAC | this study |
| SRMF1 | rrnS | 14380-14403 | fwd | AAGTAATTCTTATCGTGGACCATC | this study |
| SRRF1 | rrnS | 14672-14693 | fwd | AAGTTTAACCGCTATTGCTGGC | this study |
| SRRR1 | rrnS | 14674-14693 | rev | GCCAGCAATAGCGGTTAAAC | this study |
| ATLF1 | atr | 14924-14945 | fwd | AATGAAGTGCCTGAATAAAAGG | this study |
| ATMF1 | atr | 15174-15198 | fwd | AAATAATGCAAAACATATGCAAAAA | this study |
| ATMF3 | atr | 15508-15526 | fwd | CCAGCACGACTACCGTCTG | this study |
| ATMF2 | atr | 15575-15598 | fwd | ATTTTATTAACTGCATGAGGACAT | this study |
