# Supplementary material for: Accelerated Evolution of Mitochondrial but Not Nuclear Genomes of Hymenoptera: New Evidence from Crabronid Wasps
Source: PLoS One. 2012 Mar 6;7(3):e32826. doi: 10.1371/journal.pone.0032826 (PMC3295772; doi:10.1371/journal.pone.0032826)
Supplement: Table S2 — Primer combinations and PCR conditions used for the amplification of the mitochondrial genome of P. triangulum. (DOCX) [file pone.0032826.s004.docx]

**Table S2**: Primer combinations and PCR conditions used for the amplification of the mitochondrial genome of *P. triangulum*.

|  |  |  |  |  |  |  |  |
| --- | --- | --- | --- | --- | --- | --- | --- |
| **Region** | **Fwd primer** | **Rev**  **primer** | **Fragment length (kb)** | **Polymerase system used** | **Annealing temp. (°C)** | **Extension temp. (°C)** | **Amplification cycles** |
| cox1 | LCO | Ben | 1.1 | Sawady Taq (Peqlab) | 49 | 72 | 35 |
| cox1-cox2 | Jerry | C2LR1 | 1.0 | Mid Range PCR System (Peqlab) | 60 | 68 | 30 |
| cox1-cox2 | Jerry | Barbara_mod | 1.5 | Mid Range PCR System (Peqlab) | 60 | 68 | 30 |
| cox2 | C2LF2 | Barbara_mod | 0.4 | Sawady Taq (Peqlab) | 60 | 72 | 32 |
| cox2-cob | C2LF2 | CBLR5 | 7.9 | Mid Range PCR System (Peqlab) | 60 | 68 | 30 |
| cob-nad1 | CBRF1 | N1RR1 | 1.0 | Mid Range PCR System (Peqlab) | 60 | 68 | 30 |
| cob-rrnL | CB1 | LRLR1 | 2.1 | Mid Range PCR System (Peqlab) | 60 | 68 | 30 |
| cob-rrnL | CBRF1 | LRLR1 | 1.5 | Mid Range PCR System (Peqlab) | 60 | 68 | 30 |
| rrnL | 16Sbr | 16Sar | 0.6 | Mid Range PCR System (Peqlab) | 60 | 68 | 30 |
| rrnL-rrnS | LRLF2 | SRRR1 | 1.8 | Mid Range PCR System (Peqlab) | 60 | 68 | 30 |
| rrnL-cox1 | LRLF2 | C1LR1 | 5.1 | BIO-X-ACT Long (Bioline) | 50 | 68 | 31 |
| atr-nad2 | ATLF1 | N2LR1 | 1.6 | Expand Long Template Taq (Roche) | 55 | 60 | 30 |
| atr-nad2 | ATMF1 | N2LR1 | 1.4 | Expand Long Template Taq (Roche) | 55 | 60 | 30 |
| atr-nad2 | ATMF1 | N2LR2 | 1.3 | Expand Long Template Taq (Roche) | 55 | 60 | 30 |
| atr-nad2 | ATMF1 | N2RR1 | 1.8 | Expand Long Template Taq (Roche) | 55 | 60 | 30 |
| atr | ATMF2 | ATRR2 | 0.6 | Expand Long Template Taq (Roche) | 55 | 60 | 30 |
|  |  |  |  |  |  |  |  |
