# Supplementary material for: Accelerated Evolution of Mitochondrial but Not Nuclear Genomes of Hymenoptera: New Evidence from Crabronid Wasps
Source: PLoS One. 2012 Mar 6;7(3):e32826. doi: 10.1371/journal.pone.0032826 (PMC3295772; doi:10.1371/journal.pone.0032826)
Supplement: Table S3 — Taxonomy and GenBank accession numbers of mitochondrial genomes used for the phylogenetic analysis. (DOCX) [file pone.0032826.s005.docx]

**Table S3**: Taxonomy and GenBank accession numbers of mitochondrial genomes used for the phylogenetic analysis.

|  |  |  |  |
| --- | --- | --- | --- |
| **Species** | **Order** | **Family** | **GenBank Acc. #** |
| *Abispa ephippium* | Hymenoptera | Vespidae, Eumeninae | NC_011520 |
| *Acmaeodera sp. NCS-2009* | Coleoptera | Buprestidae | NC_013580 |
| *Acraea issoria* | Lepidoptera | Nymphalidae | NC_013604 |
| *Adelium sp. NCS-2009* | Coleoptera | Tenebrionidae | NC_013554 |
| *Adoxophyes honmai* | Lepidoptera | Tortricidae | NC_008141 |
| *Aedes albopictus* | Diptera | Culicidae | NC_006817 |
| *Anopheles gambiae* | Diptera | Culicidae | NC_002084 |
| *Anoplophora glabripennis* | Coleoptera | Cerambycidae | NC_008221 |
| *Antheraea pernyi* | Lepidoptera | Saturniidae | NC_004622 |
| *Apatides fortis* | Coleoptera | Bostrichidae | NC_013582 |
| *Apis mellifera* | Hymenoptera | Apidae, Apinae | NC_001566 |
| *Artogeia melete* | Lepidoptera | Pieridae | NC_010568 |
| *Ascaloptynx appendiculatus* | Neuroptera | Ascalaphidae | NC_011277 |
| *Aspidytes niobe* | Coleoptera | Aspidytidae | NC_012139 |
| *Bactrocera oleae* | Diptera | Tephritidae | NC_005333 |
| *Bombus ignitus* | Hymenoptera | Apidae, Bombinae | NC_010967 |
| *Bombyx mori* | Lepidoptera | Bombycidae | NC_002355 |
| *Cephus cinctus* | Hymenoptera | Cephidae | NC_012688 |
| *Ceratitis capitata* | Diptera | Tephritidae | NC_000857 |
| *Chaetosoma scaritides* | Coleoptera | Melyridae | NC_011324 |
| *Chauliognathus opacus* | Coleoptera | Cantharidae | NC_013576 |
| *Chrysochroa fulgidissima* | Coleoptera | Buprestidae | NC_012765 |
| *Chrysomya* *putoria* | Diptera | Calliphoridae | NC_002697 |
| *Cochliomyia hominivorax* | Diptera | Calliphoridae | NC_002660 |
| *Coreana raphaelis* | Lepidoptera | Lycaenidae | NC_007976 |
| *Corydalus cornutus* | Megaloptera | Corydalidae | NC_011276 |
| *Cotesia vestalis* | Hymenoptera | Braconidae | NC_014272 |
| *Crioceris duodecimpunctata* | Coleoptera | Chrysomelidae | NC_003372 |
| *Culex quinquefasciatus* | Diptera | Culicidae | NC_014574 |
| *Culicoides arakawae* | Diptera | Ceratopogonidae | NC_009809 |
| *Cydistomyia duplonotata* | Diptera | Tabanidae | NC_008756 |
| *Cyphon sp. BT0012* | Coleoptera | Scirtidae | NC_011320 |
| *Dermatobia hominis* | Diptera | Oestridae | NC_006378 |
| *Diadegma semiclausum* | Hymenoptera | Ichneumonidae | NC_012708 |
| *Diatraea saccharalis* | Lepidoptera | Crambidae | NC_013274 |
| *Ditaxis biseriata* | Neuroptera | Mantispidae | NC_013257 |
| *Drosophila melanogaster* | Diptera | Drosophilidae | NC_001709 |
| *Eriogyna pyretorum* | Lepidoptera | Saturniidae | NC_012727 |
| *Evania appendigaster* | Hymenoptera | Evaniidae | NC_013238 |
| *Haematobia irritans* | Diptera | Muscidae | NC_007102 |
| *Hipparchia autonoe* | Lepidoptera | Nymphalidae | NC_014587 |
| *Hydroscapha granulum* | Coleoptera | Hydroscaphidae | NC_012144 |
| *Hyphantria cunea* | Lepidoptera | Arctiidae | NC_014058 |
| *Hypoderma lineatum* | Diptera | Oestridae | NC_013932 |
| *Liriomyza trifolii* | Diptera | Agromyzidae | NC_014283 |

**Table S3 (continued)**: Taxonomy and GenBank accession numbers of mitochondrial genomes used for the phylogenetic analysis.

|  |  |  |  |
| --- | --- | --- | --- |
| **Species** | **Order** | **Family** | **GenBank Acc. #** |
| *Lucanus mazama* | Coleoptera | Lucanidae | NC_013578 |
| *Lucilia sericata* | Diptera | Calliphoridae | NC_009733 |
| *Lymantria dispar* | Lepidoptera | Lymantriidae | NC_012893 |
| *Macrogyrus oblongus* | Coleoptera | Gyrinidae | NC_013249 |
| *Manduca sexta* | Lepidoptera | Sphingidae | NC_010266 |
| *Mayetiola destructor* | Diptera | Cecidomyiidae | NC_013066 |
| *Melipona bicolor* | Hymenoptera | Apidae, Meliponinae | NC_004529 |
| *Mongoloraphidia harmandi* | Raphidioptera | Raphidiidae | NC_013251 |
| *Mordella atrata* | Coleoptera | Mordellidae | NC_013254 |
| *Nasonia vitripennis* | Hymenoptera | Pteromalidae | EU746609/EU746613 |
| *Neopanorpa pulchra* | Mecoptera | Panorpidae | NC_013180 |
| *Ochrogaster lunifer* | Lepidoptera | Noctuidae | NC_011128 |
| *Orussus occidentalis* | Hymenoptera | Orussidae | NC_012689 |
| *Ostrinia furnacalis* | Lepidoptera | Crambidae | NC_003368 |
| *Papilio maraho* | Lepidoptera | Papilionidae | NC_014055 |
| *Parnassius bremeri* | Lepidoptera | Papilionidae | NC_014053 |
| *Perga condei* | Hymenoptera | Pergidae | AY787816 |
| *Philaenus spumarius* | Hemiptera | Aphrophoridae | NC_005944 |
| *Philanthus triangulum* | Hymenoptera | Crabronidae | JN871914 |
| *Phthonandria atrilineata* | Lepidoptera | Geometridae | NC_010522 |
| *Polistes humilis* | Hymenoptera | Vespidae, Polistinae | EU024653 |
| *Polystoechotes punctatus* | Neuroptera | Polystoechotidae | NC_011278 |
| *Priasilpha obscura* | Coleoptera | Phloeostichidae | NC_011326 |
| *Primeuchroeus sp. M48* | Hymenoptera | Chrysididae | AH015389 |
| *Protohermes concolorus* | Megaloptera | Corydalidae | NC_011524 |
| *Psacothea hilaris* | Coleoptera | Cerambycidae | NC_013070 |
| *Pyrocoelia rufas* | Coleoptera | Lampyridae | NC_003970 |
| *Pyrophorus divergens* | Coleoptera | Elateridae | NC_009964 |
| *Radoszkowskius oculata* | Hymenoptera | Mutillidae | NC_014485 |
| *Rhagophthalmus lufengensis* | Coleoptera | Phengodidae | NC_010969 |
| *Rhopaea magnicornis* | Coleoptera | Scarabaeidae | NC_013252 |
| *Rhopalomyia pomum* | Diptera | Cecidomyiidae | NC_013063 |
| *Sasakia charonda* | Lepidoptera | Nymphalidae | NC_014224 |
| *Saturnia boisduvalii* | Lepidoptera | Saturniidae | NC_010613 |
| *Sialis hamata* | Megaloptera | Sialidae | NC_013256 |
| *Simosyrphus grandicornis* | Diptera | Syrphidae | NC_008754 |
| *Spathius agrili* | Hymenoptera | Braconidae | NC_014278 |
| *Sphaerius sp. BT0074* | Coleoptera | Sphaeriusidae | NC_011322 |
| *Spilonota lechriaspis* | Lepidoptera | Tortricidae | NC_014294 |
| *Teinopalpus aureus* | Lepidoptera | Papilionidae | NC_014398 |
| *Tetraphalerus bruchi* | Coleoptera | Ommatidae | NC_011328 |
| *Trachypachus holmbergi* | Coleoptera | Trachypachidae | NC_011329 |
| *Triatoma dimidiata* | Hemiptera | Reduviidae | NC_002609 |
| *Tribolium castaneum* | Coleoptera | Tenebrionidae | NC_003081 |
| *Trichophthalma punctata* | Diptera | Nemestrinidae | NC_008755 |
| *Vanhornia eucnemidarum* | Hymenoptera | Vanhorniidae | NC_008323 |
