# Supplementary material for: Accelerated Evolution of Mitochondrial but Not Nuclear Genomes of Hymenoptera: New Evidence from Crabronid Wasps
Source: PLoS One. 2012 Mar 6;7(3):e32826. doi: 10.1371/journal.pone.0032826 (PMC3295772; doi:10.1371/journal.pone.0032826)
Supplement: Table S4 — Taxonomy and GenBank accession numbers of nuclear genes used for the phylogenetic analysis. (DOCX) [file pone.0032826.s006.docx]

**Table S4:** Taxonomy and GenBank accession numbers of nuclear genes used for the phylogenetic analysis.

|  |  |  |  |  |
| --- | --- | --- | --- | --- |
| **Gene** | **Species** | **Order** | **Family** | **GenBank Acc. #** |
| **ArgK** | *Apis mellifera* | Hymenoptera | Apidae | NM_001011603 |
|  | *Bembidion rufotinctum* | Coleoptera | Carabidae | EF648776 |
|  | *Bombus zonatus* | Hymenoptera | Apidae | DQ788522 |
|  | *Bombyx mori* | Lepidoptera | Bombycidae | NM_001043937 |
|  | *Culex pipiens* | Diptera | Culicidae | DS231990 |
|  | *Cyphononyx dorsalis* | Hymenoptera | Pompilidae | AB264171 |
|  | *Dineutes sublineatus* | Coeloptera | Gyrinidae | EU677511 |
|  | *Drosophila melanogaster* | Diptera | Drosophilidae | DMU26940 |
|  | *Epicephala sp.* | Lepidoptera | Gracillariidae | FJ235412 |
|  | *Galapaganus ashlocki* | Coleoptera | Curculionidae | EU748600 |
|  | *Helicoverpa armigera* | Lepidoptera | Noctuidae | EF600057 |
|  | *Homalodisca coagulata* | Hemiptera | Cicadellidae | AY588062 |
|  | *Hylaeus solaris* | Hymenoptera | Colletidae | DQ212153 |
|  | *Melanocercops ficuvorella* | Lepidoptera | Gracillariidae | FJ235415 |
|  | *Nasonia vitripennis* | Hymenoptera | Pteromalidae | XM_001607072 |
|  | *Oncometopia nigricans* | Hemiptera | Cicadellidae | AY725785 |
|  | *Phyllotreta striolata* | Coleoptera | Chrysomelidae | EU420057 |
|  | *Plodia interpunctella* | Lepidoptera | Pyralidae | AJ315030 |
|  | *Solenopsis invicta* | Hymenoptera | Formicidae | EU817514 |
|  | *Stomphastis labyrinthica* | Lepidoptera | Gracillariidae | FJ235414 |
|  | *Tenomerga cinerea* | Coleoptera | Cupedidae | EU677505 |
|  | *Philanthus triangulum* | Hymenoptera | Crabronidae | JQ083477 |
|  | *Tribolium castaneum* | Coleoptera | Tenebrionidae | XM_966707 |
| **PEPCK** | *Acyrthosiphon pisum* | Hemiptera | Aphididae | XM_001949351 |
|  | *Aedes aegypti* | Diptera | Culicidae | XM_001647887 |
|  | *Agathiphaga queenslandensis* | Lepidoptera | Agathiphagidae | U28446 |
|  | *Apis mellifera* | Hymenoptera | Apidae | XM_396295 |
|  | *Baryscapus sp. CD015* | Hymenoptera | Eulophidae | DQ924426 |
|  | *Bombus pennsylvanicus* | Hymenoptera | Apidae | EF050949 |
|  | *Bombyx mori* | Lepidoptera | Bombycidae | DQ443379 |
|  | *Carabus maiyasanus* | Coleoptera | Carabidae | EU435123 |
|  | *Cardiocladius sp.* | Diptera | Chironomidae | AF047530 |
|  | *Chalcura sp. CD014* | Hymenoptera | Eucharitidae | DQ924425 |
|  | *Chauliognathus opacus* | Coleoptera | Cantharidae | EU677598 |
|  | *Copelatus distinctus* | Coleoptera | Dytiscidae | EU677603 |
|  | *Culex quinquefasciatus* | Diptera | Culicidae | XM_001859338 |
|  | *Drosophila melanogaster* | Diptera | Drosophilidae | Y00402 |
|  | *Dynastes granti* | Coleoptera | Scarabaeidae | EU677599 |
|  | *Encarsia sp. CD011* | Hymenoptera | Aphelinidae | DQ924424 |
|  | *Eriocrania semipurpurella* | Lepidoptera | Eriocraniidae | U28441 |
|  | *Gonatocerus sp. CD026* | Hymenoptera | Mymaridae | DQ924437 |
|  | *Heterobathmia pseuderiocrania* | Lepidoptera | Heterobathmiidae | U28440 |
|  | *Hockeria sp. CD023* | Hymenoptera | Chalcididae | DQ924434 |
|  | *Lymantria dispar* | Lepidoptera | Lymantriidae | U28438 |
|  | *Micropterix calthella* | Lepidoptera | Micropterigidae | U28437 |
|  | *Nasonia vitripennis* | Hymenoptera | Pteromalidae | XM_001600177 |
|  | *Platygaster sp. CD032* | Hymenoptera | Platygastridae | DQ924442 |
|  | *Porthetes hispidus* | Coleoptera | Curculionidae | EU310715 |
|  | *Scelio sp. CD036* | Hymenoptera | Scelionidae | DQ924446 |
|  | *Silpha imitator* | Coleoptera | Silphidae | AB285608 |
|  | *Simulium encisoi* | Diptera | Simuliidae | AF047541 |
|  | *Steffanolampus sp. CD040* | Hymenoptera | Perilampidae | DQ924450 |
|  | *Stenus alienus* | Coleoptera | Staphylinidae | AB285628 |
|  | *Sthenopis argenteomaculatus* | Lepidoptera | Hepialidae | U28435 |
|  | *Tenomerga cinerea* | Coleoptera | Cupedidae | EU677600 |
|  | *Thaumalea gillespieae* | Diptera | Thaumaleidae | AF047531 |
|  | *Tinea pellionella* | Lepidoptera | Tineidae | U28431 |
|  | *Tipula paterifera* | Diptera | Tipulidae | U28432 |
|  | *Tischeria citrinipennella* | Lepidoptera | Tischeriidae | U28433 |
|  | *Torymus sp. CD042* | Hymenoptera | Torymidae | DQ924452 |
|  | *Tribolium castaneum* | Coleoptera | Tenebrionidae | XM_970904 |
|  | *Trigona amazonensis* | Hymenoptera | Apidae | EF051025 |
|  | *Xylocopa auripennis* | Hymenoptera | Apidae | AY005306 |
| **wingless** | *Anopheles gambiae* | Diptera | Culicidae | AY645023 |
|  | *Apis mellifera* | Hymenoptera | Apidae | XM_396946 |
|  | *Arhopala aurea* | Lepidoptera | Lycaenidae | AY236039 |
|  | *Autosticha modicella* | Lepidoptera | Oecophoridae | EF680519 |
|  | *Bembidion levettei* | Coleoptera | Carabidae | AF398571 |
|  | *Bombus ardens* | Hymenoptera | Apidae | EU184707 |
|  | *Callomelitta antipodes* | Hymenoptera | Colletidae | EF032907 |
|  | *Carabus maiyasanus* | Coleoptera | Carabidae | EU435120 |
|  | *Chionodes stefaniae* | Lepidoptera | Gelechiidae | EF680514 |
|  | *Chyphotes sp.* | Hymenoptera | Bradynobaenida | DQ353144 |
|  | *Cicindela sedecimpunctata* | Coleoptera | Cicindelidae | AF398579 |
|  | *Clogmia albipunctata* | Diptera | Psychodidae | AY645027 |
|  | *Coboldia fuscipes* | Diptera | Scatopsidae | AY645030 |
|  | *Colias eurytheme* | Lepidoptera | Pieridae | AF537291 |
|  | *Cotesia sesamiae* | Hymenoptera | Braconidae | DQ538588 |
|  | *Diopsis apicalis* | Diptera | Diopsidae | AF304811 |
|  | *Drosophila melanogaster* | Diptera | Drosophilidae | NM_078778 |
|  | *Evagetes sp.* | Hymenoptera | Pompilidae | DQ353145 |
|  | *Feltia jaculifera* | Lepidoptera | Noctuidae | AY569039 |
|  | *Galagete darwini* | Lepidoptera | Autostichidae | EF680564 |
|  | *Grammia celia* | Lepidoptera | Arctiidae | EU333641 |
|  | *Heliconius metharme* | Lepidoptera | Heliconiidae | AF169920 |
|  | *Hesperia comma* | Lepidoptera | Hesperiidae | AY700737 |
|  | *Limenitis weidemeyerii* | Lepidoptera | Nymphalidae | EU433946 |
|  | *Lymantria dispar* | Lepidoptera | Lymantriidae | EU333627 |
|  | *Macrosoma sp.* | Lepidoptera | Hedylidae | AY569042 |
|  | *Macrosteles quadrilineatus* | Hemiptera | Cicadellidae | FJ001461 |
|  | *Microselia texana* | Diptera | Metopininae | DQ448550 |
|  | *Myrmica americana* | Hymenoptera | Formicidae | AY101371 |
|  | *Odites leucostola* | Lepidoptera | Lecithoceridae | EF680517 |
|  | *Odontophotopsis sp.* | Hymenoptera | Mutillidae | DQ353146 |
|  | *Pachypsylla celtidismamma* | Hemiptera | Psyllidae | AF231379 |
|  | *Papilio dardanus* | Lepidoptera | Papilionidae | EU157964 |
|  | *Plectroctena ugandensis* | Hymenoptera | Formicidae | EU155480 |
|  | *Pleistodontes imperialis* | Hymenoptera | Agaonidae | DQ539372 |
|  | *Stomphastis labyrinthica* | Lepidoptera | Gracillariidae | FJ235233 |
|  | *Tenebrio molitor* | Coleoptera | Tenebrionidae | EU048300 |
|  | *Tribolium castaneum* | Coleoptera | Tenebrionidae | NM_001114350 |
|  | *Trigona fuscipennis* | Hymenoptera | Apidae | EU184710 |
|  | *Venturia canescens* | Hymenoptera | Ichneumonidae | DQ538619 |
|  | *Vespula sp.* | Hymenoptera | Vespidae | DQ353143 |
|  | *Xylophanes tersa* | Lepidoptera | Sphingidae | EU479558 |
|  | *Zaprionus tuberculatus* | Diptera | Drosophilidae | DQ778974 |
