# Supplementary material for: Accelerated Evolution of Mitochondrial but Not Nuclear Genomes of Hymenoptera: New Evidence from Crabronid Wasps
Source: PLoS One. 2012 Mar 6;7(3):e32826. doi: 10.1371/journal.pone.0032826 (PMC3295772; doi:10.1371/journal.pone.0032826)
Supplement: Table S5 — Summary of the mitochondrial genome of P. triangulum. (DOCX) [file pone.0032826.s007.docx]

**Table S5**: Summary of the mitochondrial genome of *P. triangulum*.

|  |  |  |  |  |  | | |  |  |  |  |  |  |  |
| --- | --- | --- | --- | --- | --- | --- | --- | --- | --- | --- | --- | --- | --- | --- |
| **Gene** | **Start** | **End** | **Size (bp)** | **Strand** | **Distance to**  **previous (bp)^(1)^** | | | **Anticodon** | **Start codon** | **Stop codon^(2)^** |  |  |  |  |
| trnM | 1 | 67 | 67 | + | 0 | | | CAT | - | - |  |  |  |  |
| trnQ | 70 | 138 | 69 | - | 2 | | | TTG | - | - |  |  |  |  |
| nad2 | 199 | 1194 | 996 | + | 60 | | | - | ATT | TAA |  |  |  |  |
| trnC | 1193 | 1255 | 63 | - | -2 | | | GCA | - | - |  |  |  |  |
| trnY | 1257 | 1323 | 67 | - | 1 | | | GTA | - | - |  |  |  |  |
| trnW | 1330 | 1399 | 70 | + | 6 | | | TCA | - | - |  |  |  |  |
| cox1 | 1400 | 2945 | 1546 | + | 0 | | | - | ATT | T |  |  |  |  |
| trnL1 | 2946 | 3011 | 66 | + | 0 | | | TAA | - | - |  |  |  |  |
| cox2 | 3012 | 3697 | 686 | + | 0 | | | - | TTG | TA |  |  |  |  |
| trnK | 3698 | 3765 | 68 | + | 0 | | | TTT | - | - |  |  |  |  |
| trnD | 3766 | 3831 | 66 | + | 0 | | | GTC | - | - |  |  |  |  |
| atp8 | 3832 | 3996 | 165 | + | 0 | | | - | ATC | TAA |  |  |  |  |
| atp6 | 3990 | 4657 | 668 | + | -7 | | | - | ATG | TA |  |  |  |  |
| cox3 | 4658 | 5449 | 792 | + | 0 | | | - | ATG | TAA |  |  |  |  |
| trnG | 5456 | 5522 | 67 | + | 6 | | | TCC | - | - |  |  |  |  |
| nad3 | 5523 | 5875 | 353 | + | 0 | | | - | ATC | TA |  |  |  |  |
| trnA | 5876 | 5939 | 64 | + | 0 | | | TGC | - | - |  |  |  |  |
| trnR | 5949 | 6013 | 65 | + | 9 | | | TCG | - | - |  |  |  |  |
| trnN | 6017 | 6083 | 67 | + | 3 | | | GTT | - | - |  |  |  |  |
| trnE | 6084 | 6149 | 66 | + | 0 | | | TTC | - | - |  |  |  |  |
| trnS1 | 6148 | 6205 | 58 | + | -2 | | | TCT | - | - |  |  |  |  |
| trnF | 6205 | 6271 | 67 | - | -1 | | | GAA | - | - |  |  |  |  |
| nad5 | 6272 | 7956 | 1685 | - | 0 | | | - | ATT | TA |  |  |  |  |
| trnH | 7957 | 8025 | 69 | - | 0 | | | GTG | - | - |  |  |  |  |
| nad4 | 8026 | 9343 | 1318 | - | 0 | | | - | ATG | T |  |  |  |  |
| nad4L | 9337 | 9636 | 300 | - | -7 | | | - | ATT | TAA |  |  |  |  |
| trnP | 9652 | 9717 | 66 | - | 15 | | | TGG | - | - |  |  |  |  |
| trnT | 9766 | 9831 | 66 | + | 48 | | | TGT | - | - |  |  |  |  |
| nad6 | 9893 | 10431 | 539 | + | 61 | | | - | ATT | TA |  |  |  |  |
| cob | 10432 | 11577 | 1146 | + | 0 | | | - | ATA | TAA |  |  |  |  |
| trnS2 | 11584 | 11649 | 66 | + | 6 | | | TGA | - | - |  |  |  |  |
| nad1 | 11643 | 12596 | 954 | - | -7 | | | - | ATT | TAA |  |  |  |  |
| trnL2 | 12597 | 12665 | 69 | - | 0 | | | TAG | - | - |  |  |  |  |
| rrnL | 12666 | 13993 | 1328 | - | 0 | | | - | - | - |  |  |  |  |
| trnV | 13994 | 14060 | 67 | - | 0 | | | TAC | - | - |  |  |  |  |
| rrnS | 14061 | 14923 | 863 | - | 0 | | | - | - | - |  |  |  |  |
| trnI | 14924 | 14990 | 67 | + | 0 | | | GAT | - | - |  |  |  |  |
| atr | 14991 | 16029 | 1039 |  | 0 | | | - | - | - |  |  |  |  |
| **^(1)^negative distances indicate overlaps** | | | | | |  |  | |  | | |  |  |  |
| **^(2)^abbreviated stop codons (TA/T) are supposedly completed by poly-adenylation** | | | | | | | | | | | | | |  |
