# Supplementary material for: Accelerated Evolution of Mitochondrial but Not Nuclear Genomes of Hymenoptera: New Evidence from Crabronid Wasps
Source: PLoS One. 2012 Mar 6;7(3):e32826. doi: 10.1371/journal.pone.0032826 (PMC3295772; doi:10.1371/journal.pone.0032826)
Supplement: Table S6 — Nucleotide composition of the protein-coding, ribosomal RNA and transfer RNA genes in the mitochondrial genome of P. triangulum. Nucleotide frequencies for all genes were calculated for the coding strand. The strand coding for the cox1-3 genes (+) was arbitrarily chosen for the calculation of the average nucleotide frequencies of the complete genome (“All sites”). (DOCX) [file pone.0032826.s008.docx]

**Table S6**: Nucleotide composition of the protein-coding, ribosomal RNA and transfer RNA genes in the mitochondrial genome of *P. triangulum*. Nucleotide frequencies for all genes were calculated for the coding strand. The strand coding for the cox1-3 genes (+) was arbitrarily chosen for the calculation of the average nucleotide frequencies of the complete genome (“All sites”).

|  |  |  |  |  |  |  |  |
| --- | --- | --- | --- | --- | --- | --- | --- |
|  | **strand** | **sites** | **%T** | **%C** | **%A** | **%G** | **%AT** |
| All sites | + | 16029 | 39.2 | 10.2 | 44.4 | 6.1 | 83.6 |
| nad2 | + | 993 | 44.0 | 7.0 | 45.9 | 3.0 | 89.9 |
| cox1 | + | 1545 | 39.5 | 13.4 | 36.1 | 11.1 | 75.6 |
| cox2 | + | 684 | 39.9 | 12.0 | 38.3 | 9.8 | 78.2 |
| atp8 | + | 162 | 38.3 | 7.4 | 50.0 | 4.3 | 88.3 |
| atp6 | + | 666 | 43.8 | 9.6 | 39.9 | 6.6 | 83.7 |
| cox3 | + | 789 | 41.3 | 12.0 | 37.4 | 9.3 | 78.7 |
| nad3 | + | 351 | 45.9 | 8.3 | 39.9 | 6.0 | 85.8 |
| nad5 | - | 1683 | 52.6 | 5.3 | 31.9 | 10.2 | 84.5 |
| nad4 | - | 1317 | 51.6 | 4.6 | 33.8 | 10.1 | 85.4 |
| nad4L | - | 297 | 55.2 | 1.3 | 33.3 | 10.1 | 88.5 |
| nad6 | + | 537 | 43.4 | 7.4 | 46.7 | 2.4 | 90.1 |
| cob | + | 1143 | 41.5 | 13.1 | 36.9 | 8.5 | 78.4 |
| nad1 | - | 951 | 52.1 | 5.8 | 30.8 | 11.4 | 82.9 |
| Protein-coding total |  | 11118 | 45.8 | 8.6 | 36.9 | 8.7 | 82.7 |
| 1st codon positions |  | 3706 | 38.7 | 8.4 | 40.3 | 12.6 | 79.0 |
| 2nd codon positions |  | 3706 | 51.5 | 14.3 | 22.9 | 11.3 | 74.4 |
| 3rd codon positions |  | 3706 | 47.2 | 3.1 | 47.5 | 2.2 | 94.7 |
| rrnL | - | 1328 | 45.4 | 5.3 | 38.2 | 11.1 | 83.6 |
| rrnS | - | 863 | 43.2 | 4.4 | 41.0 | 11.4 | 84.2 |
| All tRNAs |  | 1460 | 41.1 | 5.6 | 45.3 | 7.9 | 86.4 |
| atr |  | 1039 | 46.2 | 8.3 | 39.5 | 6.1 | 85.7 |
