# Supplementary material for: Accelerated Evolution of Mitochondrial but Not Nuclear Genomes of Hymenoptera: New Evidence from Crabronid Wasps
Source: PLoS One. 2012 Mar 6;7(3):e32826. doi: 10.1371/journal.pone.0032826 (PMC3295772; doi:10.1371/journal.pone.0032826)
Supplement: Table S7 — Principle Components Analysis (PCA) of base composition in mitochondrial genomes of holometabolous insects. The 1st principle component accounts for most of the variability (93%) among the four nucleotides and contrasts A and T frequencies against C and G frequencies. (DOCX) [file pone.0032826.s009.docx]

**Table S7:** Principle Components Analysis (PCA) of base composition in mitochondrial genomes of holometabolous insects. The 1st principle component accounts for most of the variability (93%) among the four nucleotides and contrasts A and T frequencies against C and G frequencies.

|  | PCs | | |
| --- | --- | --- | --- |
| Eigenvectors | 1^st^ | 2^nd^ | 3^rd^ |
| A | -0.494 | -0.616 | 0.308 |
| C | 0.508 | -0.277 | -0.630 |
| G | 0.506 | 0.320 | 0.651 |
| T | -0.491 | 0.663 | -0.291 |
| Eigenvalue | 3.73 | 0.22 | 0.05 |
| Percent of variation | 93.3 | 5.4 | 1.2 |
| Cumulative percent | 93.3 | 98.7 | 100 |
|  |  |  |  |
